# Author Correction: Quantitative fragmentomics allow affinity mapping of interactomes

**DOI:** 10.1038/s41467-022-35177-6

**Published:** 2022-12-07

**Authors:** Gergo Gogl, Boglarka Zambo, Camille Kostmann, Alexandra Cousido-Siah, Bastien Morlet, Fabien Durbesson, Luc Negroni, Pascal Eberling, Pau Jané, Yves Nominé, Andras Zeke, Søren Østergaard, Élodie Monsellier, Renaud Vincentelli, Gilles Travé

**Affiliations:** 1 Équipe Labellisée Ligue 2015, Département de Biologie Structurale Intégrative, Institut de Génétique et de Biologie Moléculaire et Cellulaire (IGBMC), INSERM U1258/CNRS UMR 7104/Université de Strasbourg, 1 rue Laurent Fries, BP 10142, F-67404 Illkirch, France; 2 Institut de Génétique et de Biologie Moléculaire et Cellulaire (IGBMC), INSERM U1258/CNRS UMR 7104/Universite de Strasbourg, 1 rue Laurent Fries, BP 10142, F-67404 Illkirch, France; 3 Architecture et Fonction des Macromolécules Biologiques (AFMB), UMR 7257 CNRS-Aix-Marseille Université, Marseille, France; 4 Bioinformatics Research Group, Research Centre for Natural Sciences, Magyar tudosok korutja 2, 1117 Budapest, Hungary; 5 Novo Nordisk A/S, Global Research Technologies, Novo Nordisk Research Park, 2760 Maaloev, Denmark

**Keywords:** Protein-protein interaction networks, X-ray crystallography, Biochemical networks, Peptides

Correction to: *Nature Communications* 10.1038/s41467-022-33018-0, published online 17 September 2022

The original version of this article contained errors in Fig. 2, Fig. 4 and Fig. 5.

In the original version of Fig. 2, PCC values were missing from panels a, b and axes labels were missing from panels c, d. The correct version of Fig. 2 is:
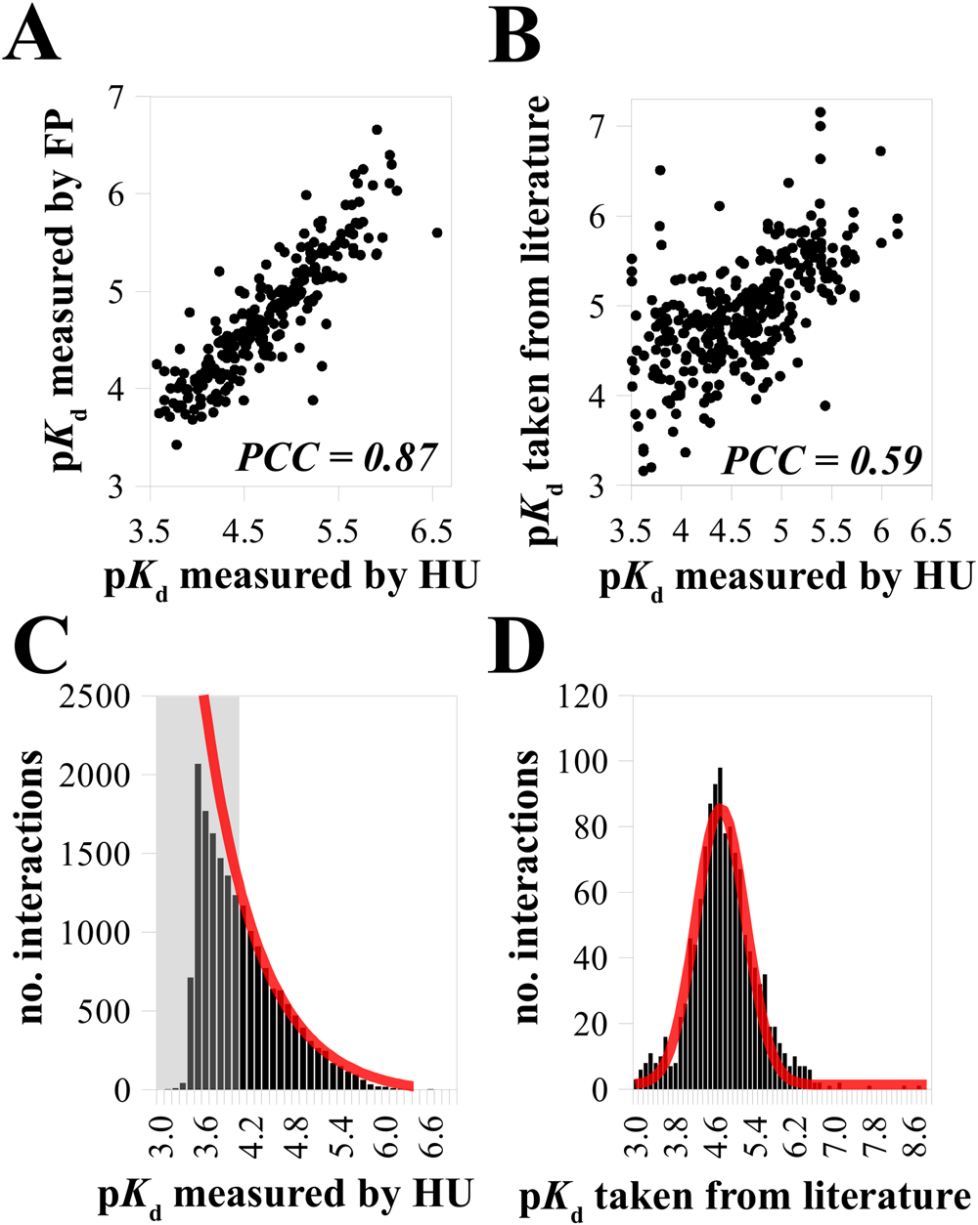


which replaces the previous incorrect version:
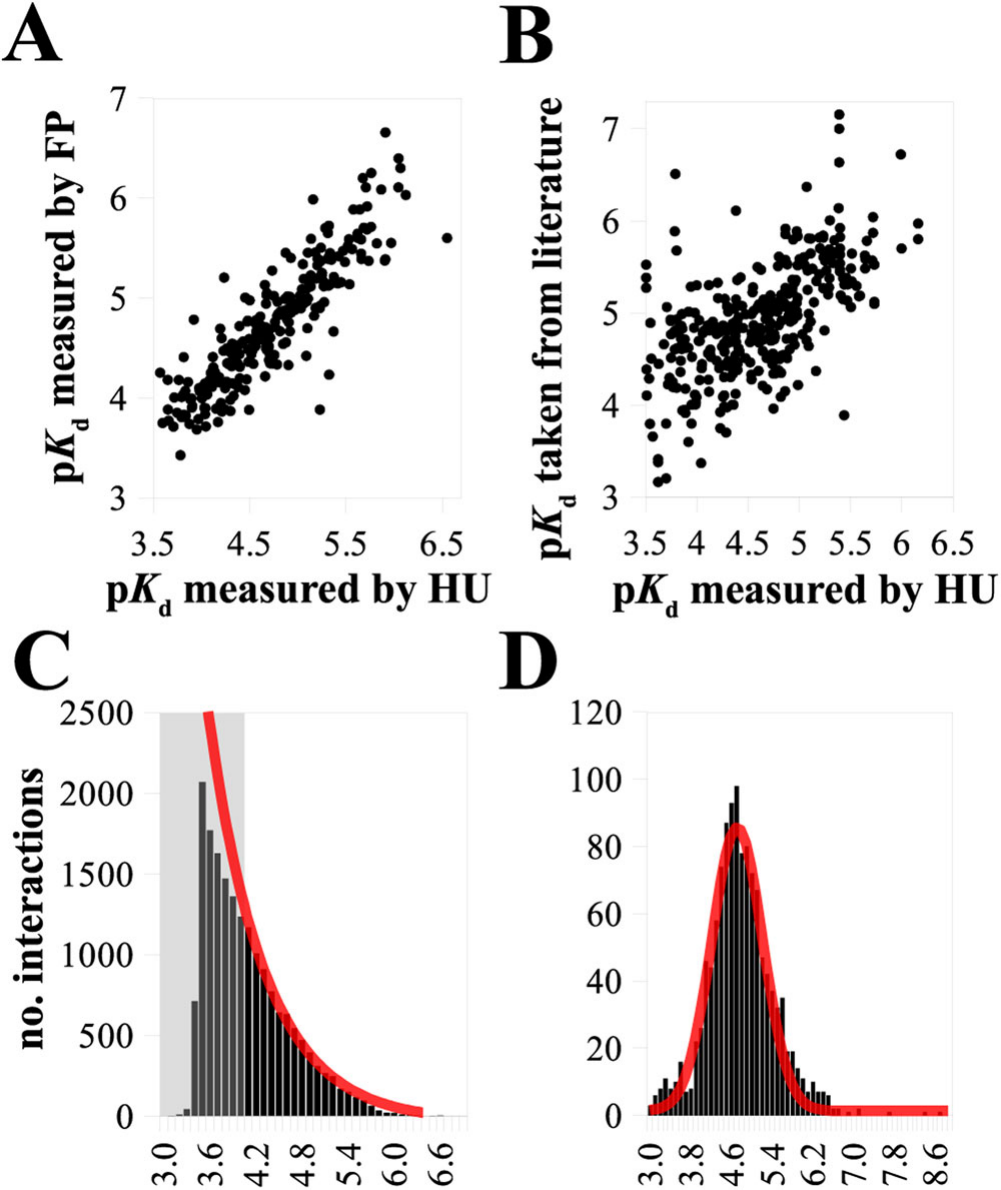


In the original version of Fig. 4, the contents of the leftmost column in panel a were missing. The correct version of Fig. 4 is:
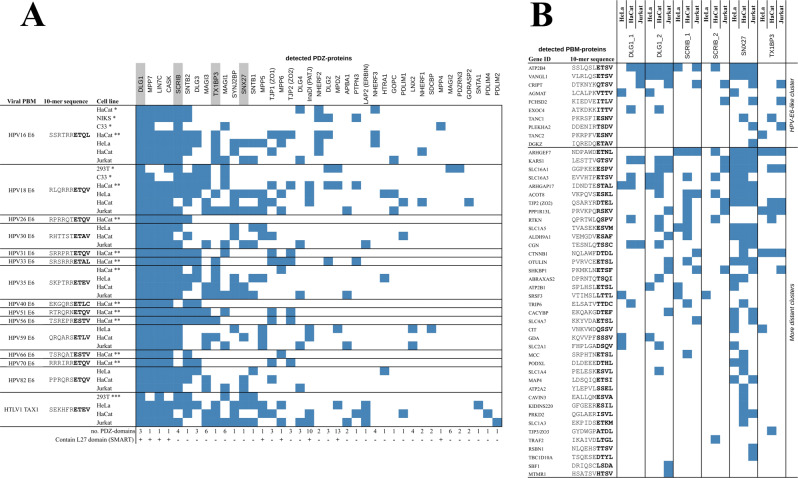


which replaces the previous incorrect version:
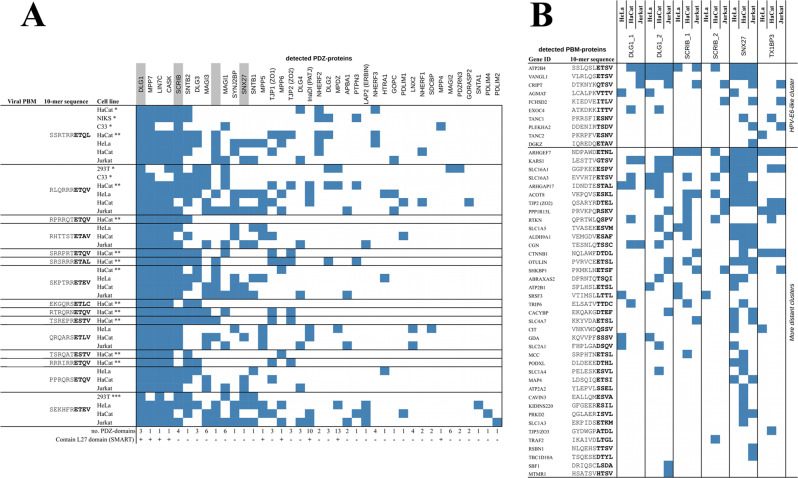


In the original version of Fig. 5, several legends as well as plot and axes labels were missing or incomplete and the order of panels did not match the descriptions in the figure caption. The correct version of Fig. 5 is:
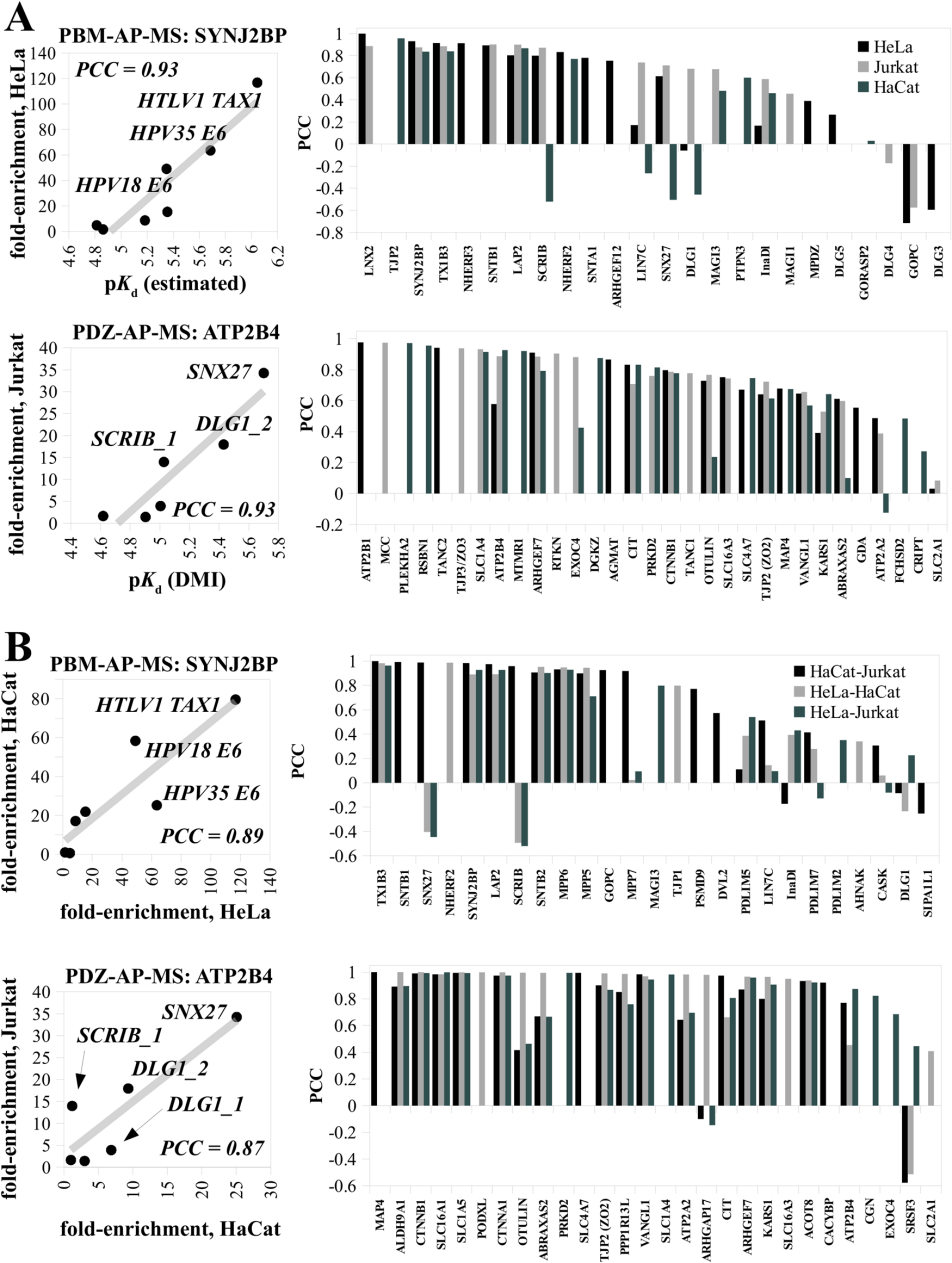


which replaces the previous incorrect version:
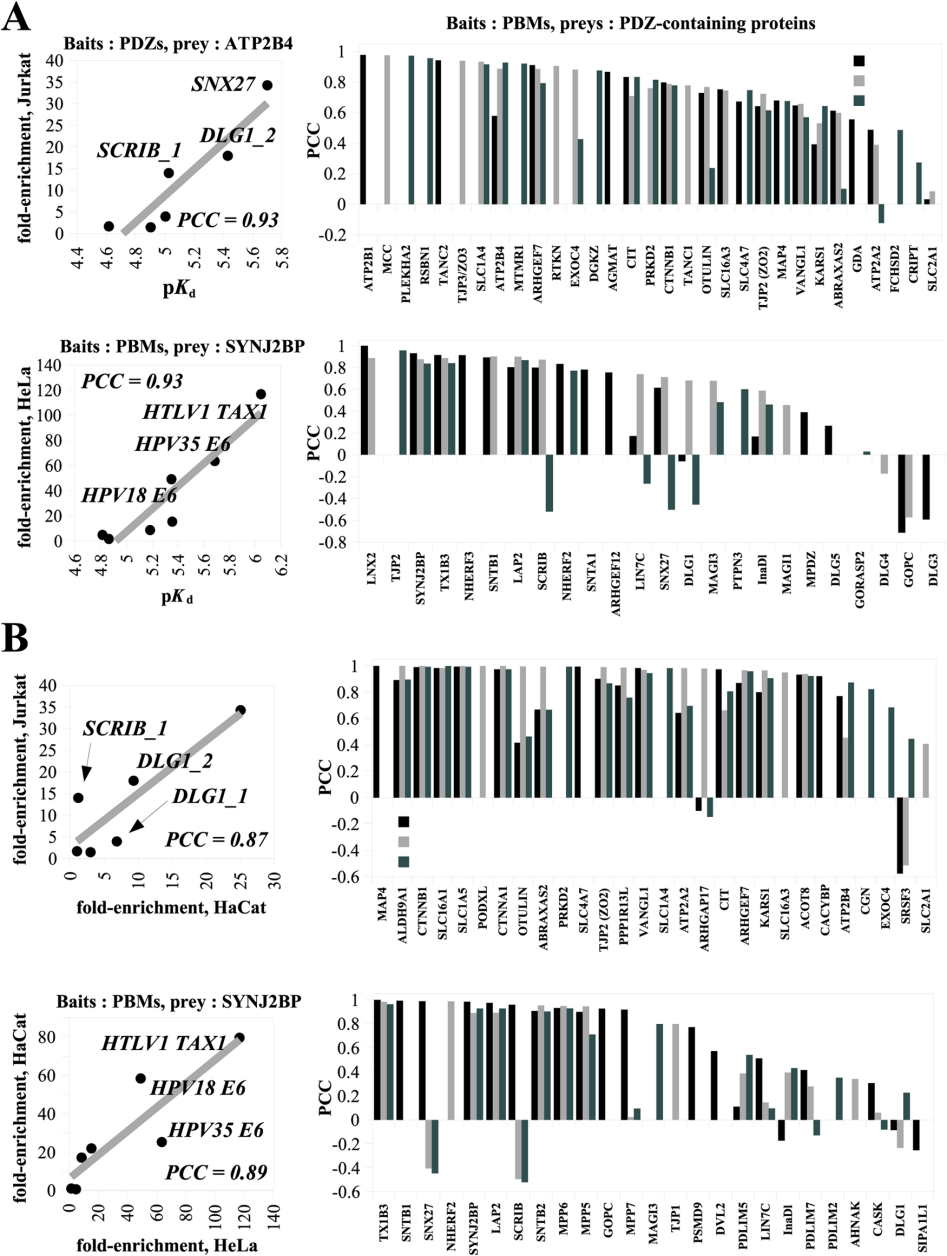


This has been corrected in both the PDF and HTML versions of the Article.

