# Supplementary material for: Quantitative fragmentomics allow affinity mapping of interactomes
Source: Nat Commun. 2022 Sep 17;13:5472. doi: 10.1038/s41467-022-33018-0 (PMC9482650; doi:10.1038/s41467-022-33018-0)
Supplement: Supplementary file 1 — Supplementary Information [file 41467_2022_33018_MOESM1_ESM.pdf]

	SNTB1 + HPV35-E6	MAGI1_2 + HPV35-E6	SNX27 + MERS-E	SYNJ2BP + HTLV1-TAX1	SYNJ2BP + RPS6KA1_-3P
**Wavelength (Å)**	1.00	1.00	1.00	0.98	0.98
**Resolution range (Å)**	43.22 - 2.0 (2.05 – 2.00)	47.64 - 2.6 (2.67 - 2.60)	44.99 - 2.15 (2.20 - 2.15)	46.68 - 1.85 (1.90 - 1.85)	46.71 - 1.9 (1.95 - 1.9)
**Space group**	$P\ 2_1\ 2_1\ 2_1$	C 1 2 1	$P\ 2_1\ 2_1\ 2_1$	$P\ 2_1\ 2_1\ 2_1$	$P\ 2_1\ 2_1\ 2_1$
**Unit cell [a, b, c (Å)]**	54.8, 60.58, 140.64	192.13, 61.03, 98.99	61.17, 83.95, 106.59	60.45, 63.99, 146.95	59.62, 75.15, 108.46
**[α, β, γ (º)]**	90, 90, 90	90, 97.334, 90	90, 90, 90	90, 90, 90	90, 90, 90
**Total reflections**	428453 (32415)	149517 (10968)	404120 (30110)	662292 (50179)	519462 (39686)
**Unique reflections**	32358 (2327)	33417 (2471)	30598 (2226)	48765 (3522)	39032 (2825)
**Multiplicity**	13.2 (13.9)	4.5 (4.4)	13.2 (13.5)	13.6 (14.2)	13.3 (14.0)
**Completeness (%)**	99.5 (98.7)	94.4 (94.5)	99.9 (100)	98.3 (97.5)	99.5 (98.8)
**Mean I/sigma(I)**	12.57 (1.36)	19.06 (1.69)	8.97 (1.34)	15.18 (1.75)	11.16 (1.46)
**R-meas**	15.3 (213.3)	5.0 (111.5)	25.2 (226.0)	14.2 (229.2)	17.0 (255.0)
**CC1/2**	99.9 (50.3)	100.0 (85.2)	99.8 (71.9)	99.9 (59.3)	99.9 (59.3)
**Reflections used in refinement**	32298	33122	30509	48700	38968
**Reflections used for R-free**	1612	1641	1529	2434	1949
**R-work**	0.1826	0.2427	0.1991	0.1687	0.1810
**R-free**	0.2086	0.2841	0.2397	0.1940	0.2107
**Number of atoms**	3566	6701	3530	3931	3675
**macromolecules**	3296	6635	3259	3479	3427
**ligands**	17	51	17	31	16
**solvent**	253	15	254	421	232
**Protein residues**	414	854	409	426	422
**RMS(bonds)**	0.003	0.004	0.002	0.005	0.003
**RMS(angles)**	0.55	0.88	0.44	0.71	0.56
**Ramachandran favored (%)**	97.56	97.28	97.76	97.87	97.84
**Ramachandran allowed (%)**	2.20	2.48	2.00	1.90	1.92
**Ramachandran outliers (%)**	0.24	0.24	0.25	0.24	0.24
**Rotamer outliers (%)**	1.97	3.10	1.41	2.64	0.81
**Clashscore**	3.14	4.60	2.28	2.56	2.32
**Average B-factor**	46.13	103.23	47.20	38.70	46.27
**macromolecules**	45.97	103.22	47.11	37.87	46.10
**ligands**	45.07	113.68	50.51	44.99	43.07
**solvent**	48.32	68.24	48.07	45.10	48.93
**PDB ID**	**7P70**	**7P71**	**7P72**	**7P73**	**7P74**

Supplementary Table 1,

Supplementary Table 1

X-Ray Data refinement statitistics.

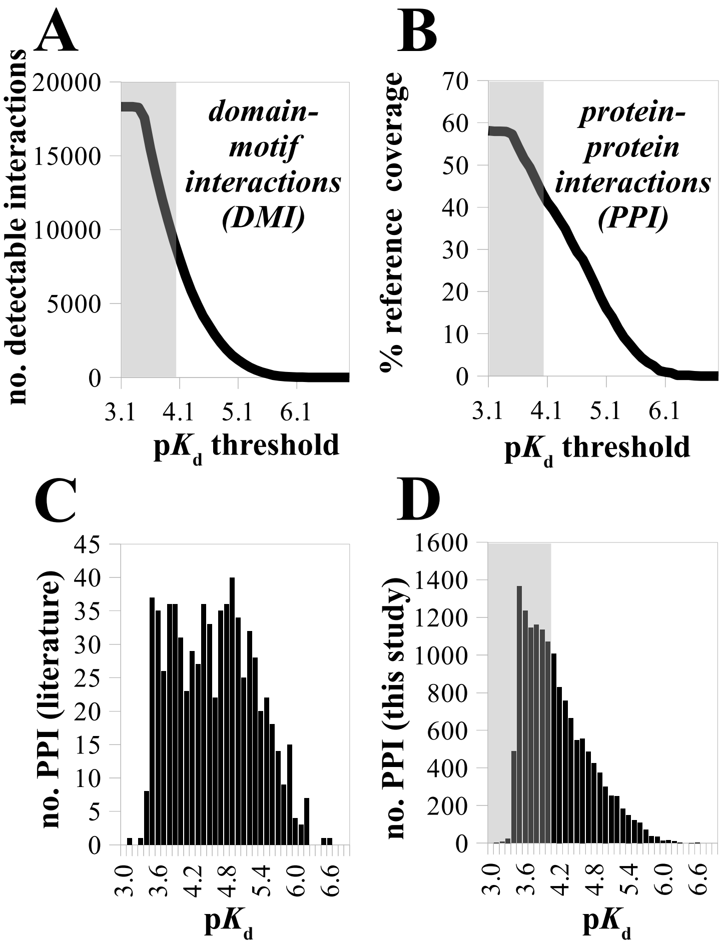

Supplementary Figure 1

Supplementary Figure 1

Comparing the quantitative PDZ-PBM affinities measured in our work to qualitative interactions from the literature.

(A) Number of PDZ-PBM interactions detected by our screen, that would have been retained by other methods with different sensitivity thresholds. In our dataset, we have used, by default, the lowest possible pK_d quantification thresholds (situated in the grey zone on the plot) derived from the binding detection thresholds of the source holdup data. This way, we quantified the dissociation constants of 18,332 out of 65,157 measured interactions. The curve describes the number of these interactions that would have been retained using less sensitive methods, as a function of the minimal detection threshold p$K_{d\,min}$ of those methods. For instance, a method with p$K_{d\,min}$ = 5 would only detect the ~1500 interactions displaying a K_d < 10 μM. (B) Coverage by our data of previously known protein-protein interactions, as a function of sensitivity threshold. For the interactomic region explored by our dataset, we plotted the percentage of qualitative protein-protein interactions (PPI) documented in large scale interactomic resources (n=1,248) which found a match to PDZ-PBM pK_d values quantified in our measurements, in the function of a variable minimal detection threshold p$K_{d\,min}$, defined as before. For proteins with multiple PDZ domains, we assumed an "additive" effect between the distinct binding sites (see Methods). If we account for all the interactions detected and quantified in our assay, our data confirm nearly 60% of the interactions (direct or indirect) reported in large scale resources. If we consider that our measurements could have been performed using less sensitive methods, this percentage further decreases, as a function of the p$K_{d\,min}$ assumed for these methods. Source data are provided in the Source Data file. (C) Affinity distribution histogram of all the protein-protein interactions found in large scale interactomic resources, for which we could match PDZ-PBM pK_d values quantified in our measurements. The affinities used for the distribution are taken from our dataset. For interactions involving PDZ-proteins with multiple PDZ-domains, "additive" affinities were considered. Histograms were calculated using all available affinities with a bin size of 0.1 pK_d. (D) Affinity distribution histogram of all the potential human protein-protein interactions that may exist according to our domain-motif data. The affinities used for the distribution are obtained from our PDZ-PBM affinity dataset. For interactions involving PDZ-proteins with multiple PDZ-domains, "additive" affinities were considered.

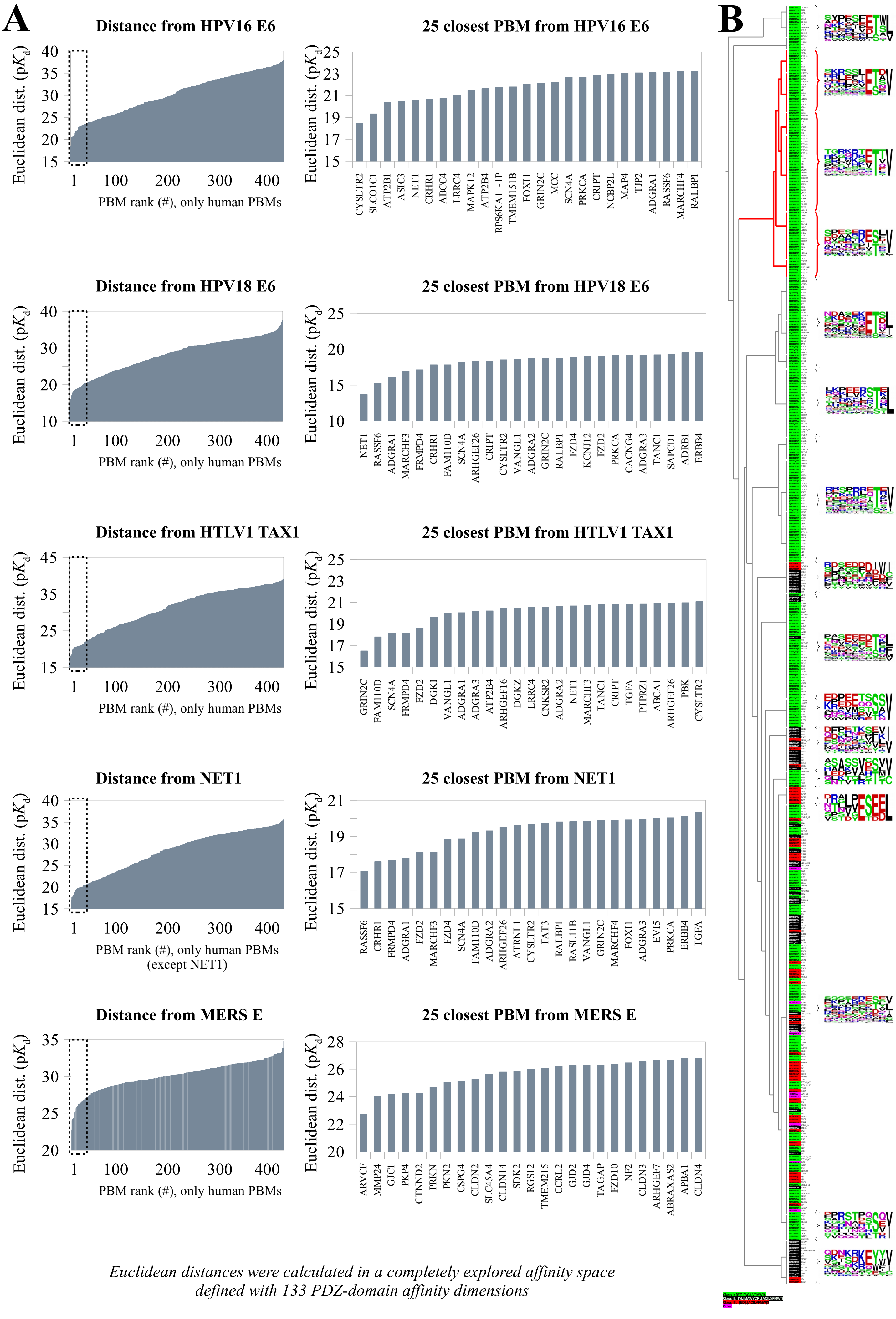
A
Distance from HPV16 E6
25 closest PBM from HPV16 E6
Euclidean dist. (pKd)
PBM rank (#), only human PBMs
CYSLTR2 SLCO1C1 ATP2B1 ASIC3 NET1 CRHR1 ABCC4 LRRC4 MAPK12 ATP2B4 RPS6KA1_-1P TMEM151B FOXI1 GRIN2C MCC SCN4A PRKCA CRIPT NCBP2L MAP4 TJP2 ADGRA1 RASSF6 MARCHF4 RALBP1
Distance from HPV18 E6
25 closest PBM from HPV18 E6
NET1 RASSF6 ADGRA1 MARCHF3 FRMPD4 CRHR1 FAM110D SCN4A ARHGEF26 CRIPT CYSLTR2 VANGL1 ADGRA2 GRIN2C RALBP1 FZD4 KCNJ12 FZD2 PRKCA CACNG4 ADGRA3 TANC1 SAPCD1 ADRB1 ERBB4
Distance from HTLV1 TAX1
25 closest PBM from HTLV1 TAX1
GRIN2C FAM110D SCN4A FRMPD4 FZD2 DGKI VANGL1 ADGRA1 ADGRA3 ATP2B4 ARHGEF16 DGKZ LRRC4 CNKSR2 ADGRA2 NET1 MARCHF3 TANC1 CRIPT TGFA PTPRZ1 ABCA1 ARHGEF26 PBK CYSLTR2
Distance from NET1
25 closest PBM from NET1
PBM rank (#), only human PBMs (except NET1)
RASSF6 CRHR1 FRMPD4 ADGRA1 FZD2 MARCHF3 FZD4 SCN4A FAM110D ADGRA2 ARHGEF26 ATRNL1 CYSLTR2 FAT3 RALBP1 RASL11B VANGL1 GRIN2C MARCHF4 FOXI1 ADGRA3 EVI5 PRKCA ERBB4 TGFA
Distance from MERS E
25 closest PBM from MERS E
ARVCF MMP24 GJC1 PKP4 CTNND2 PRKN PKN2 CSPG4 CLDN2 SLC45A4 CLDN14 SDK2 RGS12 TMEM215 CCRL2 GJD2 GJD4 TAGAP FZD10 NF2 CLDN3 ARHGEF7 ABRAXAS2 APBA1 CLDN4
B
Euclidean distances were calculated in a completely explored affinity space defined with 133 PDZ-domain affinity dimensions

Supplementary Figure 2

Finding interactomic neighborhoods in the explored Euclidean affinity space.

(A) Closest neighbors of a few selected motifs. Eucledian distance profiles are shown for HPV16 E6, HPV18 E6, HTLV1 Tax1, NET1, and MERS E. On the right panel, a zoomed in view is shown of the closest 25 motifs. Note that the unexplored part of the PBMome may contain other motifs with more similar binding properties to these references. Source data are provided as a Source Data file. (B) According to their quantified affinities with 133 common PDZ domains, PBMs were clustered based on their Euclidean distances using an UPGMA algorithm (see Methods). The identified cladogram is shown along with the sequences of motifs. Sequences are colored according to their conventional classification (PBMs of class 1, 2 and 3 in green, black and red, respectively; internal or atypical PBMs in purple). The clades enriched in oncoviral PBMs is highlighted in bold red. Sequence based frequency logos are shown for all major clusters.

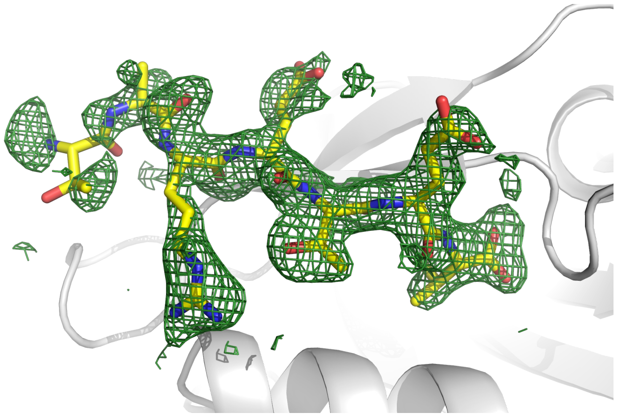

SNTB1 bound to HPV35 E6
(SA omit F_o-F_c map @ 2 σ)

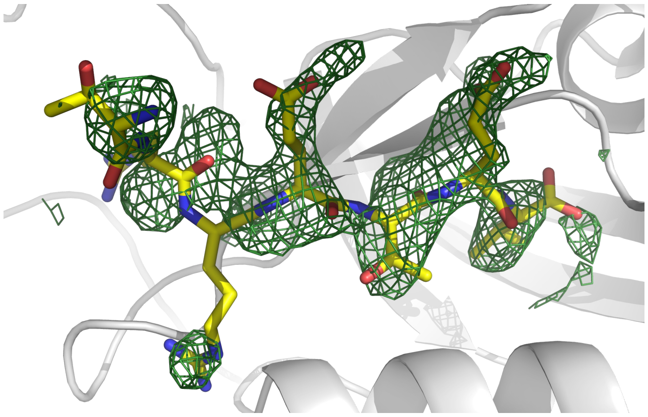

MAGI1_2 bound to HPV35 E6
(SA omit F_o-F_c map @ 2 σ)

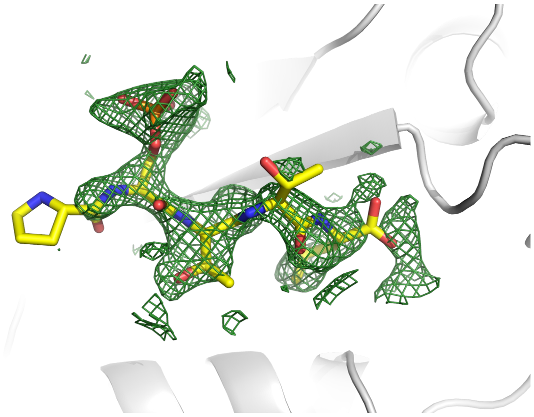

SYNJ2BP bound to phos. RSK1
(SA omit F_o-F_c map @ 2 σ)

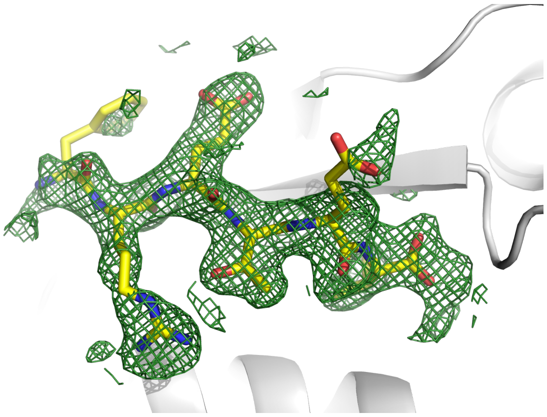

SYNJ2BP bound to HTLV1 TAX1
(SA omit F_o-F_c map @ 2 σ)

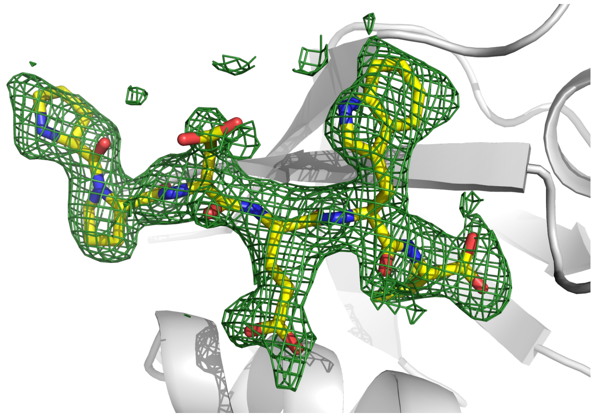

SNX27 bound to MERS E
(SA omit F_o-F_c map @ 2 σ)

Supplementary Figure 3

Supplementary Figure 3

Crystallographic omit maps for peptide-bound PDZ domain structures.

A

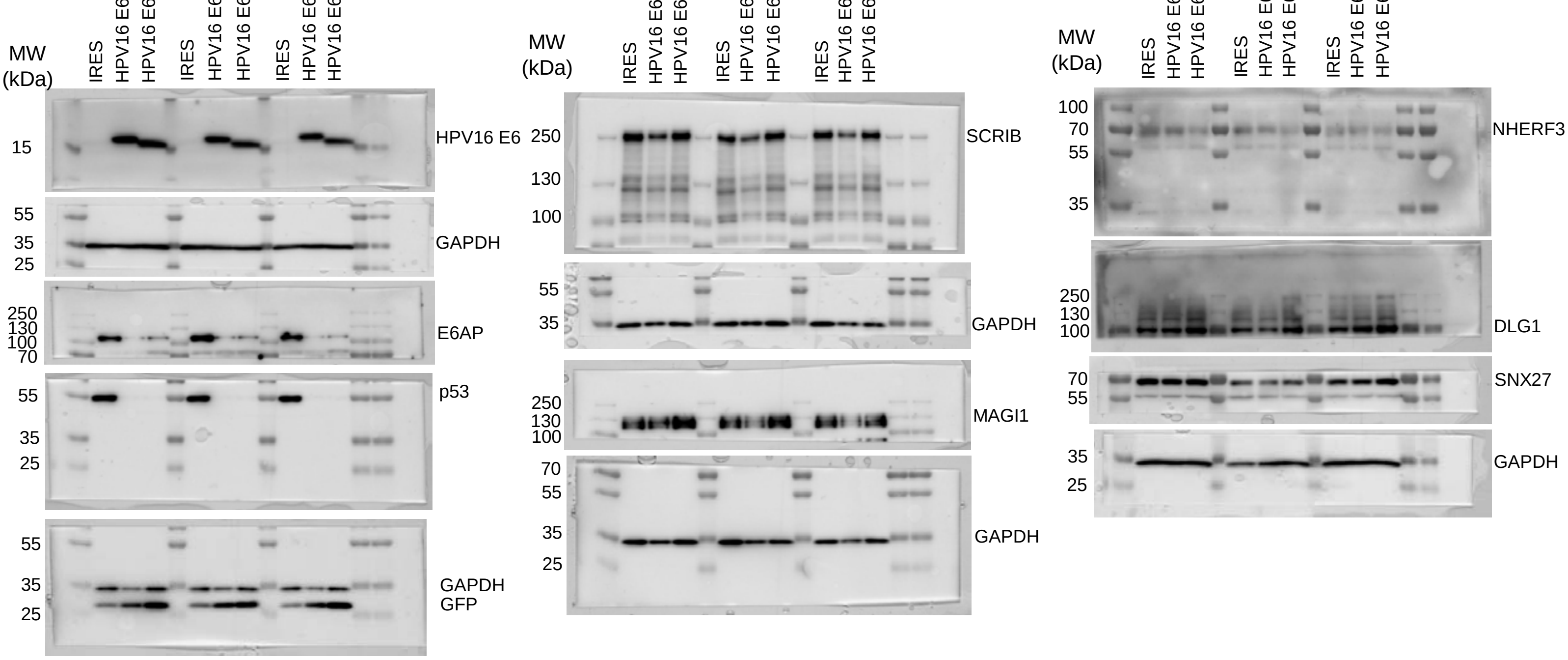

B

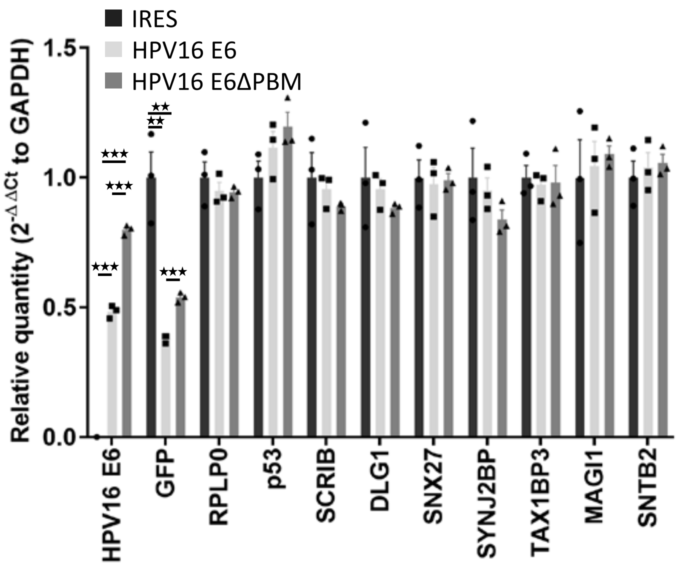

Name	Sequence
GAPDH_F	GTCTCCTCTGACTTCAACAGC
GAPDH_R	CCACCCTGTTGCTGTAGCC
HPV16-E6_F	CAGTTACTGCGACGTGAGGT
HPV16-E6_R	CCTGTCCACCGACCCCTTAT
GFP_F	GAGCGCACCATCTTCTTCAAG
GFP_R	TGTCGCCCTCGAACTTCAC
RPLP0_F	TGGTCATCCAGCAGGTGTTCGA
RPLP0_R	ACAGACACTGGCAACATTGCGG
P53_F	CCTCAGCATCTTATCCGAGTGG
P53_R	TGGATGGTGGTACAGTCAGAGC
SCRIB_F	CATCTCTTCCATCGACCGGG
SCRIB_R	CACTAGGGGAGCAGGGAGAT
DLG1_F	AGAGCAACCTCTTTCAGGCT
DLG1_R	CTGCTTGGCAGTGTCTCTGA
SNX27_F	GCATCGTCAAGTCCGAGTCC
SNX27_R	CTCGAATCAGGTCCACCACC
SYNJ2BP_F	GGCTCCAGGAGGGTGATAAG
SYNJ2BP_R	GTTGCCGGTATCTCATGAAAGC
TAX1BP3_F	AGAGCAGGGTCGAGATGTCC
TAX1BP3_R	TCACCTGCATGATCTTGTCTCC
MAGI1_F	AGATCACTCAGCCCTTGTGC
MAGI1_R	CAGGTTCATCCCCTCCAACC
SNTB2_F	GAGGACTCTGGTTCGCCAAA
SNTB2_R	GTGGCTGTATCTTTGCAGCG

C

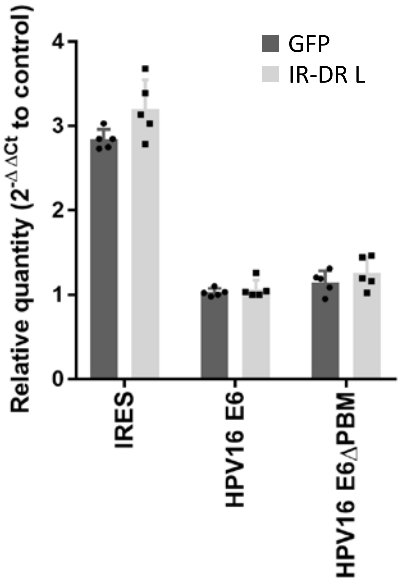

Name	Sequence
RPPH1_F	AGCTGAGTGCGTCCTGTCACT
RPPH1_R	TCTGGCCCTAGTCTCAGACCTT
GFP_F	GAGCGCACCATCTTCTTCAAG
GFP_R	TGTCGCCCTCGAACTTCAC
IRDRL_F	CTCGTTTTTCAACTACTCCACAAATTTCT
IRDRL_R	GTGTCATGCACAAAGTAGATGTCCTA

Supplementary Figure 4

Validation of 293T cell lines stably expressing HPV16 E6 and HPV16 E6ΔPBM.

(A) Analysis of some PDZ protein expressions in these cells by Western-blot. (B) Top: RT-qPCR analysis of the RNA expression of different proteins in these cells (mean ± SD, 3 biological replicates). Two-tailed unpaired t-tests were used to determine statistical significance (*: $p<0.05$, **: $p<0.01$, ***: $p<0.001$). Exact p-values are provided together with the other source data in the source file. Bottom: Primers used for the RT-qPCR analyses. (C) Top: Relative copy numbers (mean ± SD, 5 biological replicates) of 293T cell lines stably expressing HPV16 E6 and HPV16 E6ΔPBM. Bottom: Primers used for copy number determination.
