# Supplementary material for: Quantitative fragmentomics allow affinity mapping of interactomes
Source: Nat Commun. 2022 Sep 17;13:5472. doi: 10.1038/s41467-022-33018-0 (PMC9482650; doi:10.1038/s41467-022-33018-0)
Supplement: Supplementary file 3 — Description_of_Additional_Supplementary_Files [file 41467_2022_33018_MOESM3_ESM.doc]

**Description of Additional Supplementary Files:**

File name: Supplementary Data 1. Description: Affinity survey and benchmarking of the PDZ-PBM interactome.

File name: Supplementary Data 2. Description: Results of the AP-MS experiments and their analysis.

File name: Supplementary Data 3. Description: Results of proteomic characterization 293T cells stably expressing HPV16 E6, HPV16 E6ΔPBM, or the empty vector (IRES).

The contents of the three files are more precisely described and explained in the first sheet of each file, called “Legend”.
