# Supplementary material for: Quantitative fragmentomics allow affinity mapping of interactomes
Source: Nat Commun. 2022 Sep 17;13:5472. doi: 10.1038/s41467-022-33018-0 (PMC9482650; doi:10.1038/s41467-022-33018-0)
Supplement: Supplementary file 7 — Reporting Summary [file 41467_2022_33018_MOESM7_ESM.pdf]

nature portfolio

Corresponding author(s): Gergo Gogl, Gilles Trave

Last updated by author(s): Feb 2, 2022

Reporting Summary

Nature Portfolio wishes to improve the reproducibility of the work that we publish. This form provides structure for consistency and transparency in reporting. For further information on Nature Portfolio policies, see our Editorial Policies and the Editorial Policy Checklist.

Statistics

For all statistical analyses, confirm that the following items are present in the figure legend, table legend, main text, or Methods section.

n/a	Confirmed	
☐	☒	The exact sample size (*n*) for each experimental group/condition, given as a discrete number and unit of measurement
☐	☒	A statement on whether measurements were taken from distinct samples or whether the same sample was measured repeatedly
☐	☒	The statistical test(s) used AND whether they are one- or two-sided<br>*Only common tests should be described solely by name; describe more complex techniques in the Methods section.*
☒	☐	A description of all covariates tested
☐	☒	A description of any assumptions or corrections, such as tests of normality and adjustment for multiple comparisons
☐	☒	A full description of the statistical parameters including central tendency (e.g. means) or other basic estimates (e.g. regression coefficient) AND variation (e.g. standard deviation) or associated estimates of uncertainty (e.g. confidence intervals)
☐	☒	For null hypothesis testing, the test statistic (e.g. *F*, *t*, *r*) with confidence intervals, effect sizes, degrees of freedom and *P* value noted<br>*Give P values as exact values whenever suitable.*
☒	☐	For Bayesian analysis, information on the choice of priors and Markov chain Monte Carlo settings
☒	☐	For hierarchical and complex designs, identification of the appropriate level for tests and full reporting of outcomes
☐	☒	Estimates of effect sizes (e.g. Cohen's *d*, Pearson's *r*), indicating how they were calculated

Our web collection on statistics for biologists contains articles on many of the points above.

Software and code

Policy information about availability of computer code

Data collection	Holdup: PheraStar control software, MARS; Mass spectrometry: Proteome Discoverer 2.4 software (PD2.4, Thermo Fisher Scientific)
Data analysis	Crystallography: XDS (version 2021), Phenix (version 1.14-3260), Phaser (part of Phenix version 1.14-3260); Fluorescence Polarization: ProFit (version 1); Holdup analysis: Excel (version 2019), Wester Blot image analysis: Fiji ImageJ (version 1.53c); Interactomic database: ProfAff (version 1, https://github.com/GoglG/ProfAff)

For manuscripts utilizing custom algorithms or software that are central to the research but not yet described in published literature, software must be made available to editors and reviewers. We strongly encourage code deposition in a community repository (e.g. GitHub). See the Nature Portfolio guidelines for submitting code & software for further information.

Data

Policy information about availability of data

All manuscripts must include a data availability statement. This statement should provide the following information, where applicable:

- Accession codes, unique identifiers, or web links for publicly available datasets
- A description of any restrictions on data availability
- For clinical datasets or third party data, please ensure that the statement adheres to our policy

www.profaff.igbmc.science (source code deposited at the https://github.com/GoglG/ProfAff address)
Crystal structures were deposited to PDB with IDs: 7P70, 7P71, 7P72, 7P73, 7P74
Mass spectometry data is deposited to PRIDE with ID: PXD027743

Field-specific reporting

Please select the one below that is the best fit for your research. If you are not sure, read the appropriate sections before making your selection.

- [x] Life sciences
- [ ] Behavioural & social sciences
- [ ] Ecological, evolutionary & environmental sciences

For a reference copy of the document with all sections, see nature.com/documents/nr-reporting-summary-flat.pdf

Life sciences study design

All studies must disclose on these points even when the disclosure is negative.

Sample size	Only highly robust biochemical and cell culture experiments were performed and we used experimental triplicates (cell based experiments and fluorescence polarization) or singlicates (holdup) according to the robustness of the assay based on extensive validation detailed in the manuscript.
Data exclusions	No data were excluded from the analysis.
Replication	All cell culture based experiments were performed minimum 3 times independently. All attempts at replication were successful.<br>In biochemical assays routine replicates were used - when applicable - based on previous findings and experiments were repeated using proteins from independent purifications over period of years.
Randomization	This is not relevant for our study. Our study did not involve experiments where randomization would increase the reliability of the results.
Blinding	This is not relevant for our study. Our study did not involve experiments where blinding would increase the reliability of the results.

Reporting for specific materials, systems and methods

We require information from authors about some types of materials, experimental systems and methods used in many studies. Here, indicate whether each material, system or method listed is relevant to your study. If you are not sure if a list item applies to your research, read the appropriate section before selecting a response.

Materials & experimental systems

n/a	Involved in the study
☐	☒ Antibodies
☐	☒ Eukaryotic cell lines
☒	☐ Palaeontology and archaeology
☒	☐ Animals and other organisms
☒	☐ Human research participants
☒	☐ Clinical data
☒	☐ Dual use research of concern

Methods

n/a	Involved in the study
☒	☐ ChIP-seq
☒	☐ Flow cytometry
☒	☐ MRI-based neuroimaging

Antibodies

Antibodies used	anti-GAPDH (Merck #MAB374 clone 6C5), anti-GFP (clone 2A3, IGBMC polyclonal), anti-HPV16 E6 (IGBMC polyclonal 6F4, recognizing the N-terminus of the protein), anti-p53 (CST clone 7F5 #2527), anti-E6AP (Sigma clone 3E5 #SAB1404508-100UG), anti-SCRIB (Thermo Fisher #PA5-54821), anti-MAGI1 (Santa Cruz #sc-100326), anti-SAP97/DLG1 (Thermo Fisher #PA1-741), anti-SNX27 (Thermo Fisher #MA5-27854), anti-PDZK1/NHERF3 (Santa Cruz #sc-100337), HRP conjugated Affinipure goat anti-mouse(H+L) (Jackson Immunoresearch #115-035-146), HRP conjugated Affinipure goat anti-rabbit(H+L) (Jackson Immunoresearch #111-035-003)
Validation	https://www.merckmillipore.com/FR/fr/product/Anti-Glyceraldehyde-3-Phosphate-Dehydrogenase-Antibody-clone-6C5,MM_NF-MAB374<br>anti-GFP 2A3 antibody: raised in the IGBMC antibody platform, validated in several articles from the institute (PMIDs: e.g. 22121025, 23917616, 20016065)<br>anti-HPV16 E6 6F4 antibody: raised in the IGBMC antibody platform, validated in several previous articles of the authors (PMIDs: 10398405, 11570842, 16507364, 12867640)<br>https://www.cellsignal.com/products/primary-antibodies/p53-7f5-rabbit-mab/2527<br>https://www.sigmaaldrich.com/FR/en/product/sigma/sab1404508<br>https://www.thermofisher.com/antibody/product/SCRIB-Antibody-Polyclonal/PA5-54821<br>https://www.scbt.com/p/magi-1-antibody-ss-5<br>https://www.thermofisher.com/antibody/product/SAP97-Antibody-Polyclonal/PA1-741<br>https://www.thermofisher.com/antibody/product/SNX27-Antibody-clone-1C6-Monoclonal/MA5-27854<br>https://www.scbt.com/p/pdzk1-antibody-f-36<br>https://www.jacksonimmuno.com/catalog/products/115-035-146

https://www.jacksonimmuno.com/catalog/products/111-035-003

Eukaryotic cell lines

Policy information about cell lines

Cell line source(s)	All cell lines (HEK293T, HeLa, Jurkat, HaCat) were obtained from the cell culture platform of IGBMC
Authentication	Jurkat and HaCat cell lines were not authenticated (original source: ECACC and Deutsches Krebsforschungszentrum DKFZ, respectively). HEK293T and HeLa cells were authenticated and found 100% identity to ATCC cat. CRL-3216 and ATCC cat. CCL-2, respectively.
Mycoplasma contamination	All cell lines are constantly monitored for mycoplasma contamination at the IGBMC cell culture platform. All cell lines were tested negative for Mycoplasma contamination prior to experiments.
Commonly misidentified lines (See ICLAC register)	Misidentification was not reported for these cell lines.
